# Supplementary material for: Assessment of genome packaging in AAVs using Orbitrap-based charge-detection mass spectrometry
Source: Mol Ther Methods Clin Dev. 2021 Nov 29;24:40–7. doi: 10.1016/j.omtm.2021.11.013 (PMC8671526; doi:10.1016/j.omtm.2021.11.013)
Supplement: Document S1. Supplemental materials and methods, Figures S1–S4, and Table S1 [file mmc1.pdf]

OMTM, Volume *24*

Supplemental information

Assessment of genome packaging in AAVs using Orbitrap-based charge-detection mass spectrometry

Tobias P. Wörner, Joost Snijder, Olga Friese, Thomas Powers, and Albert J.R. Heck

Supplementary Text

Optimizing conditions for the quantitative analysis of rAAV particles; pressure dependence

The pressures in different sections of a mass spectrometer are important parameters in native MS experiments and must be balanced delicately to ensure a successful experimental outcome [1–3]. Although generally high vacuum conditions are important in mass spectrometry, a somewhat elevated pressure, allowing collisional cooling, enhances effective transmission of high mass ions species through the mass spectrometer [4,5]. On the other hand, collisions with background gas can cause ion decay, and lead to eFT (enhanced or absorption mode FT) artefacts resulting is peak splitting in the acquired signals causing a systematic trailing to lower intensities in Orbitrap based CD-MS [6–8]. We illustrate this effect in Supplemental Figure 1A, where we used increasing gas pressure settings and investigate its effect on the transmission of different AAV6a capsid variants. As more often used in native MS we introduced Xenon as collision- and cooling gas. While for the lowest pressure setting, we observed barely any trailing to lower intensities, the transmission of the filled rAAV capsid particles at 28,000 *m/z* became significantly reduced. However, at elevated pressure settings, the transmission of the full rAAV species increased but at the same time, the frequency of peak splitting increased as well, requiring an additional filtering step in the downstream data analysis. The results of this peak splitting and filtering on the final mass histograms are depicted in Supplemental Figure 1B. In the unfiltered data, we observe some density trailing to lower masses (at around 3 MDa) associated with a reduced peak intensity of the split peaks. These "low-mass"-artifacts increase the FWHM of the corresponding distribution and cause a slight underestimation of its corresponding average mass. Additionally, as higher mass ions seem to be more prone to peak splitting, such centroids will be removed more frequently in the signal filtering step, biasing the quantification of filled and empty capsids and furthermore, reducing sensitivity for high mass, low abundant ion species. These artifacts can be mitigated by reducing somewhat the transient recording time from 1024 to 512 ms, as demonstrated previously [9]. The effect of a shortened transient time on the mass histogram is also shown in Supplemental Figure 1C, where we can see little to no

difference between the unfiltered and filtered datasets, when recording for 512 ms. The slightly reduced charge resolution, caused by the shorter transient length, increases the FWHM of the mass histogram for the empty capsids from 232 kDa to 246 kDa for the unfiltered data (see also Supplemental Figure 1B&C). This can be considered insignificant considering the wide inherent mass distribution of AAV (>150 kDa FWHM) and the additional peak broadening caused by solvent adducts as well as the fact that the full and empty AAV can still be resolved in the 2D histogram. The reduced transient length allows us to access a much wider pressure range which we examined for the ratio of full and empty as well as potential top mass reduction as illustrated in Supplemental Figure 1d. From these analyses we define a Xenon pressure setting between 1.5 to 3 to give the best balance between optimal and even transmission of all rAAV particles, reducing as much as possible artefacts induced by ion decay processes due to interactions with the neutral gas molecules. Therefore, we decided to use a Xenon pressure setting of 2 and 3 for the analysis of the rAAV preparations. The conversion for the used pressure setting to the resulting cold cathode gauge readout are shown in Supplemental Figure 1E.

Supplementary References

1. Boeri Erba, E., Signor, L., and Petosa, C. (2020). Exploring the structure and dynamics of macromolecular complexes by native mass spectrometry. J. Proteomics *222*, 103799.

2. Laganowsky, A., Reading, E., Hopper, J.T.S., and Robinson, C. V. (2013). Mass spectrometry of intact membrane protein complexes. Nat. Protoc. *8*, 639–651.

3. Tahallah, N., Pinkse, M., Maier, C.S., and Heck, A.J.R. (2001). The effect of the source pressure on the abundance of ions of noncovalent protein assemblies in an electrospray ionization orthogonal time-of-flight instrument. Rapid Commun. Mass Spectrom. *15*, 596–601.

4. van de Waterbeemd, M., Fort, K.L., Boll, D., Reinhardt-Szyba, M., Routh, A., Makarov, A., and Heck, A.J.R. (2017). High-fidelity mass analysis unveils heterogeneity in intact ribosomal particles. Nat. Methods *14*, 283–286.

5. Van Den Heuvel, R.H.H., Van Duijn, E., Mazon, H., Synowsky, S.A., Lorenzen, K., Versluis, C., Brouns, S.J.J., Langridge, D., Van Der Oost, J., Hoyes, J., et al. (2006). Improving the performance of a quadrupole time-of-flight instrument for macromolecular mass spectrometry. Anal. Chem. *78*, 7473–7483.

6. Sanders, J.D., Grinfeld, D., Aizikov, K., Makarov, A., Holden, D.D., and Brodbelt, J.S. (2018). Determination of Collision Cross-Sections of Protein Ions in an Orbitrap Mass Analyzer. Anal. Chem. *90*, 5896–5902.

7. Wörner, T.P., Snijder, J., Bennett, A., Agbandje-McKenna, M., Makarov, A.A., and Heck, A.J.R. (2020). Resolving heterogeneous macromolecular assemblies by Orbitrap-based single-particle charge detection mass spectrometry. Nat. Methods *17*, 395–398.

8. Lange, O., Damoc, E., Wieghaus, A., and Makarov, A. (2014). Enhanced Fourier transform for Orbitrap mass spectrometry. Int. J. Mass Spectrom. *369*, 16–22.

9. Wörner, T.P., Aizikov, K., Snijder, J., Fort, K.L., Makarov, A.A., and Heck, A.J.R. (2021). Frequency chasing of individual megadalton ions in an Orbitrap analyzer improves precision of analysis in single molecule mass spectrometry. bioRxiv, 2021.06.15.448530.

Supplementary Figures

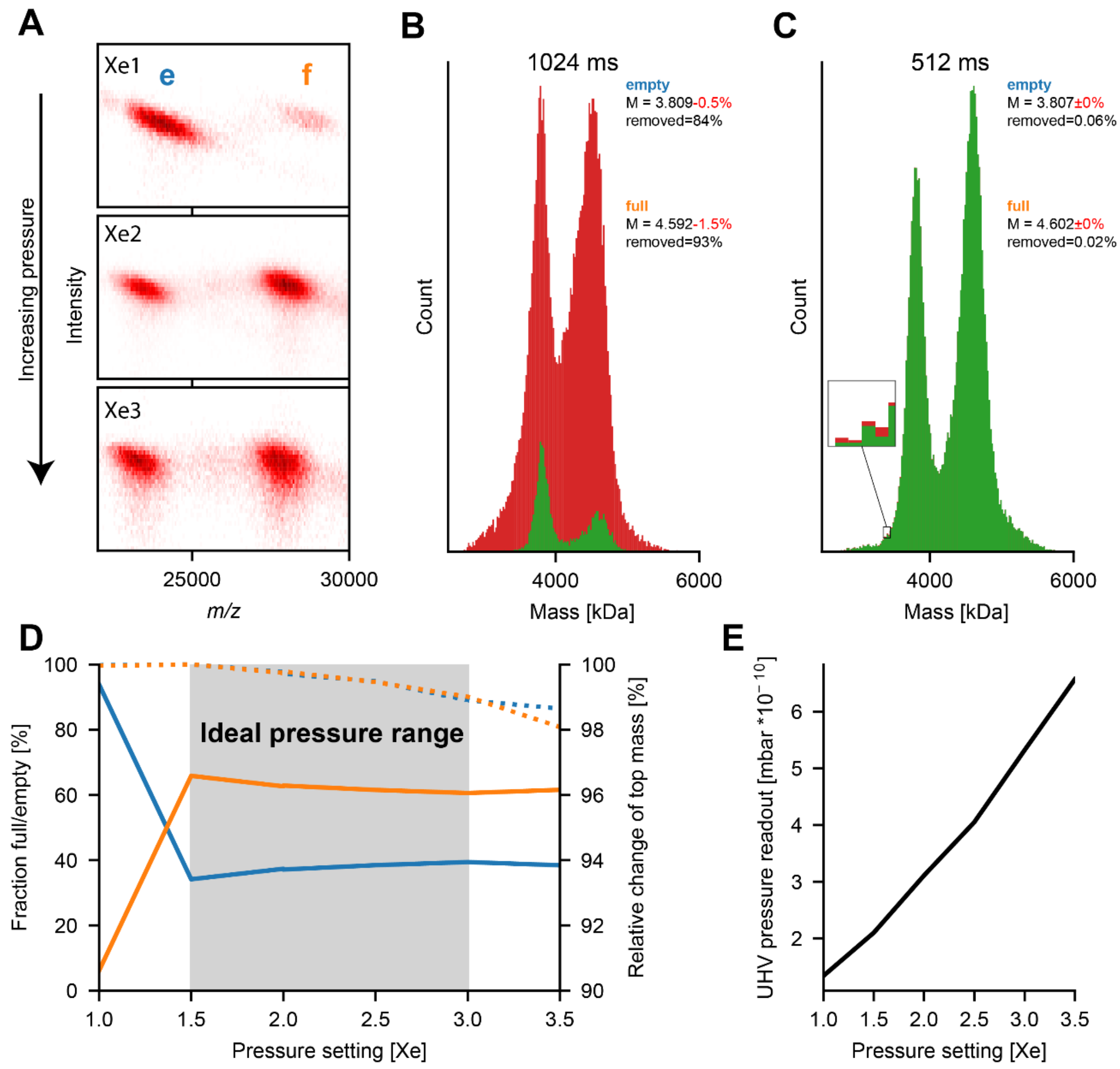

Supplemental Figure 1: Optimizing pressure and transient times for the unbiased and sensitive analysis of co-occurring rAAV particles. A) 2D histogram of mixture of empty (**e**) and filled (**f**) rAAV capsids recorded at different pressure settings, illustrating the effect of background gas on the transmission as well as peak splitting. While the filled particles are hardly transmitted at lower pressure, compared to the empty species, elevated background pressure offers a flatter transmission profile but also increased peak splitting at transient times of ~1024 ms. **B and C)**, Influence of split peak filtering on AAV capsid mixtures recorded at different transient times of 1024 ms and 512 ms (unfiltered and filtered mass histogram are shown in red and green respectively). The number of removed centroids as well as the assigned mass, including the change with respect to the unfiltered dataset, are indicated for each distribution. For the longer transient time (**B**) extensive peak splitting takes place, resulting in significant trailing towards lower masses for the unfiltered data (red). This trailing causes a reduced effective resolving power as well as a slight underestimation of the assigned masses. The required filtering recovers the suppressed resolution (R=16.3 to R=18.8 for empty capsid) and top mass but removes a significant portion of the recorded data and suppresses low abundant signal, especially in the higher mass range (>5MDa). For the shorter transient time (**C**) peak splitting is reduced significantly removing the need for filtering in general (R=15.5 for empty capsid). **D)** Tested pressure range for optimal transmission of full and empty particles at ~0.5s transients time without filtering. Pressure setting between 1.5 and 3 show flat transmission profile for empty and filled particles while still only exhibiting insignificant amounts of split peak artifacts (reduced top mass shown as dotted lines). **E)** Relation between experimental pressure setting and UHV read-out pressure.

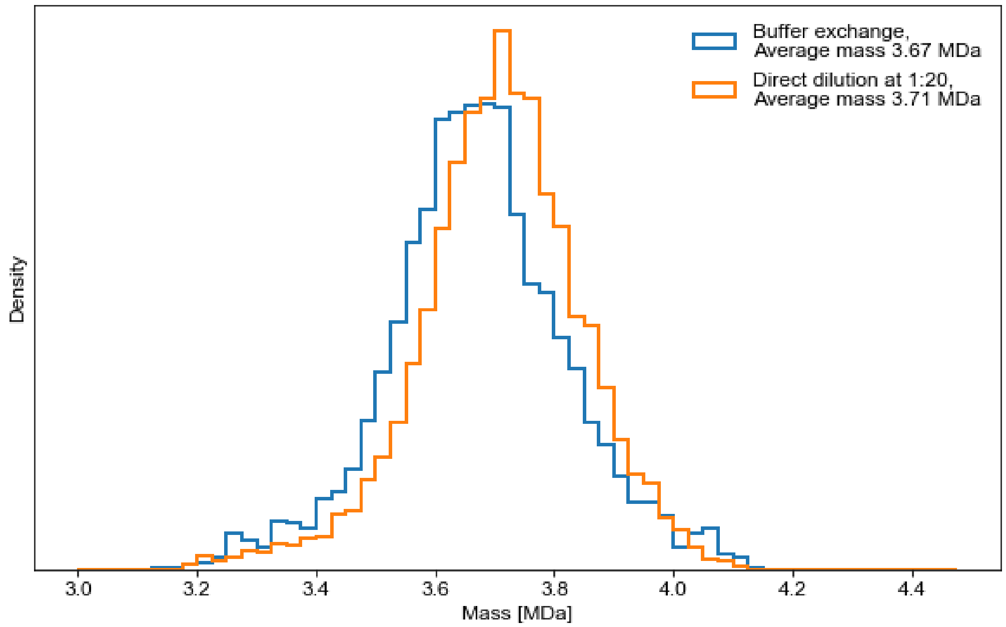

Supplemental Figure 2: Comparison between buffer exchange and direct dilution. Mass histogram of ions produced from AAV6b, which were either buffer exchanged using concentrators (blue) or directly diluted dilution into ammonium acetate with a dilution factor of 20 (orange). The average mass difference is less than 1% (36 kDa) and no significant peak broadening can be observed due to salt adducts.

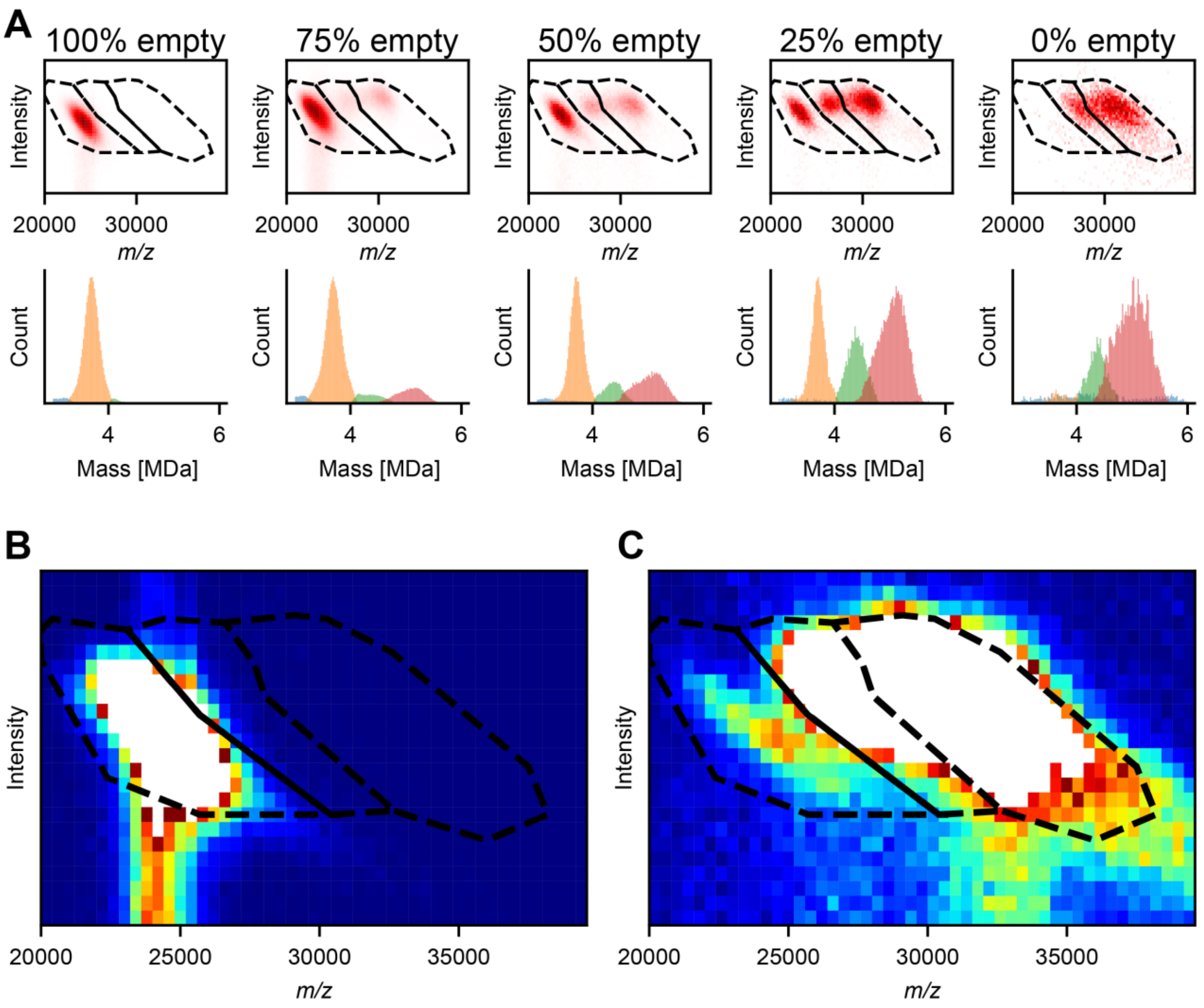

Supplemental Figure 3: Quantification of distinct AAV6b particles at elevated pressure. A) Applied AAV quantification workflow on fractionated and mixed AAV particles at Xenon pressure setting 3. 2D histogram and corresponding mass histograms for a 20 minutes dataset of the seized particles mixed at defined ratios (amount of empty fraction indicated above). **B and C)** Pooled datasets of seized empty and full fraction displayed with the filtering masked used for quantification. The empty fraction (**B**) does not show any signs of filled particles, however we do observe a small amount of signal, leaking into the assigned margins for intermediate particles. For the filled particles (**C**) we can observe a clear, low-abundant distribution of empty particles (~4%), responsible for the systematic overestimation of the empty particles in Figure 4C, also highlighting the challenges associated with rAAV purification.

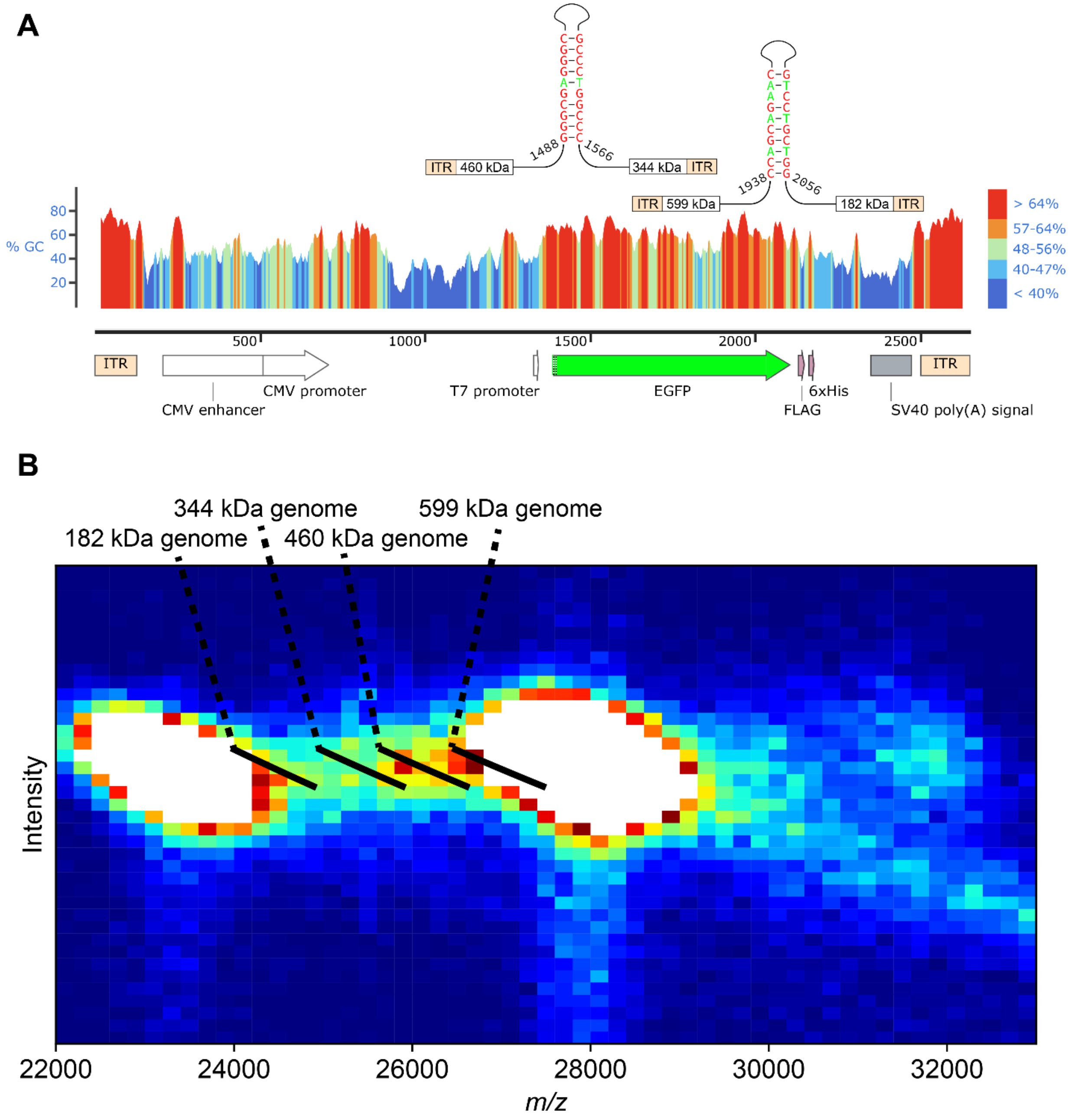

Supplemental Figure 4: Detection of low abundant rAAV variants caused by heterogeneity in the packed DNA. A) The transgene cassette to be packed into a rAAV exhibits several G/C-rich (red) regions attributed to ITRs but also in the CMV promoter as well as in the EGFP sequence. The EGFP transgene contains particular regions of palindromic G/C-rich sequences where genome replication might be impaired. The expected genome weight is indicated for a replication termination of the corresponding sense or antisense strand. **B)** 2D Histogram taken from Figure 2A with altered color coding emphasizing the occurrence of low abundant species. The expected position of a capsid encapsulating the potential truncated genomes in **A)** coincide with the detected signals between the empty and filled rAAV particles.

Supplementary Table

Supplemental Table 1: Quantification accuracy. The first column provides the expected % based on the mixing of the "full" and empty particles. The measured % of empty AAV6b capsids at pressure settings of Xe2 and Xe3 are shown in column 2 and 3. The average deviation is calculated based on the two experiments. In column 4 a correction is made based on the fact that the "full" fraction also contains about 4% "empty particles" as revealed by the CD-MS data. The overall average deviation is 3% and 1.3% (before and after correction for the contaminated full fraction)

Expected % empty AAV6b from mixing [%]	Measured empty AAV6b at Xe2 [%]	Measured empty AAV6b at Xe3 [%]	After correction [%]	Average deviation after correction [%]
75	78	76	76	1
50	53	56	52	2.5
25	23	27	28	0.5
